# Choice of dialysis modality: patients’ experiences and quality of decision after shared decision-making

**DOI:** 10.1186/s12882-020-01956-w

**Published:** 2020-08-05

**Authors:** Jeanette Finderup, Kirsten Lomborg, Jens Dam Jensen, Dawn Stacey

**Affiliations:** 1 Department of Renal Medicine, Aarhus University Hospital, Palle Juul-Jensens Boulevard 99, 8200 Aarhus, Aarhus N Denmark; 2 Department of Clinical Medicine, Aarhus University, Aarhus, Denmark; 3 School of Nursing, University of Ottawa, Ottawa, Canada; 4 Clinical Epidemiology Program, Ottawa Hospital Research Institute, Ottawa, Canada

**Keywords:** Dialysis choice, Shared decision-making, Patient decision aid, Mixed method study

## Abstract

**Background:**

Patients with kidney failure experience a complex decision on dialysis modality performed either at home or in hospital. The options have different levels of impact on their physical and psychological condition and social life. The purpose of this study was to evaluate the implementation of an intervention designed to achieve shared decision-making for dialysis choice. Specific objectives were: 1) to measure decision quality as indicated by patients’ knowledge, readiness and achieved preferences; and 2) to determine if patients experienced shared decision-making.

**Method:**

A mixed methods descriptive study was conducted using both questionnaires and semi-structured interviews. Eligible participants were adults with kidney failure considering dialysis modality. The intervention, based on the Three-Talk model, consisted of a patient decision aid and decision coaching meetings provided by trained dialysis coordinators. The intervention was delivered to 349 patients as part of their clinical pathway of care. After the intervention, 148 participants completed the Shared Decision-Making Questionnaire and the Decision Quality Measurement, and 29 participants were interviewed. Concordance between knowledge, decision and preference was calculated to measure decision quality. Interview transcripts were analysed qualitatively.

**Results:**

The participants obtained a mean score for shared decision-making of 86 out of 100. There was no significant difference between those choosing home- or hospital-based treatment (97 versus 83; *p* = 0.627). The participants obtained a knowledge score of 82% and a readiness score of 86%. Those choosing home-based treatment had higher knowledge score than those choosing hospital-based treatment (84% versus 75%; *p* = 0.006) but no significant difference on the readiness score (87% versus 84%; *p* = 0.908). Considering the chosen option and the knowledge score, 83% of the participants achieved a high-quality decision. No significant difference was found for decision quality between those choosing home- or hospital-based treatment (83% versus 83%; *p* = 0.935). Interview data informed the interpretation of these results.

**Conclusions:**

Although there was no control group, over 80% of participants exposed to the intervention and responded to the surveys experienced shared decision-making and reached a high-quality decision. Both participants who chose home- and hospital-based treatment experienced the intervention as shared decision-making and made a high-quality decision. Qualitative findings supported the quantitative results.

**Trial registration:**

The full trial protocol is available at ClinicalTrials. Gov (NCT03868800). The study has been registered retrospectively.

## Background

Involving the patient in making the decision on dialysis choice has been recommended internationally for a decade [1] but is difficult to implement in clinical practice [2–4]. Factors impeding implementation in practice include lack of time before initiating dialysis, lack of power by the patient to engage in the decision-making process, not offering patients a one-to-one discussion with their healthcare professional and incomplete or biased information on the options [2–4].

Patient decision aids (PDAs) are effective interventions to increase patient involvement in making health decisions [5]. After using a PDA, patients are more knowledgeable, clearer about their values, more involved in decision-making and more likely to make values-based decisions after using a PDA. PDAs facilitate shared decision-making (SDM) defined as:

*An approach where clinicians and patients share the best available evidence when faced with the task of making decisions, and where patients are supported to consider options, to achieve informed preferences* [6]*.*

In 2018, nine studies evaluating interventions based on SDM in dialysis choice were identified [7], but few had been rigorously evaluated in clinical practice. In fact, none of these studies evaluated the SDM process or the decisional outcomes.

In general, there is a lack of evidence regarding how best to implement PDAs and SDM in clinical practice [8]. It seems that interventions that target both patients and healthcare professionals improve implementation of SDM in clinical practice. Of the nine studies evaluating interventions for dialysis choice, five targeted both the patient and the healthcare professional [7].

According to the definition of SDM given above, an intervention should support patients to make an informed decision based on their own preferences. This is consistent with the two criteria from the International Patient Decision Aids Standards (IPDAS) for evaluating SDM interventions:

*1) The intervention should help the patient to know about the available options and their features. 2) The intervention should improve the match between the features that matter most to the informed patient and the option that is chosen* [9].

This study was conducted as part of a larger research project to evaluate an intervention to support patients with chronic kidney disease who were considering dialysis options. The intervention is called SDM and Dialysis Choice (SDM-DC) and consists of a PDA called Dialysis Choice and meetings with a dialysis coordinator that focus on: a) creating an understanding of why a choice is being made and which options there are to choose between; b) providing insight into the options as well as discussion of the advantages and disadvantages of each option; and c) making a decision. The SDM-DC intervention was pilot tested with 137 patients at one Danish hospital and was shown to be acceptable to use by healthcare professionals in an encounter with patients [7]. The pilot test finished before this evaluation study.

The purpose of this study was to evaluate the implementation of the SDM-DC intervention within four Danish hospitals. Specific objectives were: 1) to measure shared decision making and decision quality as indicated by patients’ knowledge, readiness and achieved preferences; and 2) to determine if patients experienced shared decision-making. The second objective was embedded within a concurrently conducted qualitative study to explore patients’ experiences. Full study details are reported elsewhere [10].

## Methods

### Design

A descriptive evaluation was chosen, with a focus on implementation within clinical practice, given that there is little evidence on implementation of SDM interventions. Most PDAs as SDM interventions have been evaluated in trials and then not used after the trial has been completed [11]. A concurrent convergent and embedded mixed method design was used [12, 13]. First, quantitative data was collected using standardized questionnaires. This was followed by qualitative data collection using semi-structured individual interviews based on the quantitative data. The analyses of the quantitative and qualitative data were conducted separately then integrated in the interpretation phase. An advisory board consisting of six dialysis coordinators and two patients were involved in the whole research process, including interpretation of the research findings, to ensure the findings would be relevant to end users [14–16].

### Setting

From a total of 14 hospitals with dialysis facilities in Denmark, the SDM intervention was used at four hospitals. These hospitals are within three of the country’s five healthcare regions. The intervention was developed and pilot tested from August 2015 to September 2016 at one of the four hospital. The pilot hospital and the three others then volunteered to implement the intervention as part of routine care. One is a university hospital treating 10% of all patients with kidney failure in Denmark, and the three others are regional hospitals covering 4, 5 and 7% respectively [17]. Patients with kidney failure choose between home- and hospital-based dialysis. Home-based options are haemodialysis and peritoneal dialysis that can be managed solely by the patient and his or her family. Peritoneal dialysis may also be managed with support from a healthcare professional visiting the patient’s home. The hospital-based treatment option is haemodialysis provided by a healthcare professional. All facilities encourage their patients to attend a two-day ‘kidney school’ where each day involves a four-hour information session on chronic kidney disease.

### Participants

From October 2016 to May 2018, all adult patients with kidney failure referred to a department of renal medicine at one of the four hospitals were offered the SDM-DC intervention and invited to participate in the study. The inclusion criteria were an eGFR below 20 ml/min and clinical judgement of the contact doctor or nurse indicating declining eGFR. Exclusion criteria were patients who had decided on conservative management (i.e., no dialysis), patients with a set date for transplantation using a living donor, and patients not able to participate in the intervention due to cognitive impairment. The use of an interpreter was not an exclusion criterion.

### Intervention

The SDM-DC intervention was designed for patients with kidney failure who must make a decision regarding type of dialysis: haemodialysis or peritoneal dialysis. SDM-DC consists of giving a PDA called Dialysis Choice to the patient and his or her relative(s), and meeting(s) with a dialysis coordinator. The patient and his or her family could view optional videos describing other patients’ experiences of making this decision. The decision about dialysis modality was made by the patient and his or her relatives together with the dialysis coordinator. The dialysis coordinator documented the decision in each patient’s electronic health record. Patients and healthcare professionals were involved in the development of the intervention [7].

The intervention was developed based on the Three-Talk model [6]. The purpose of the Three-Talk model is to apply SDM in clinical practice. In the model, SDM is described as three key steps: choice talk, option talk and decision talk. The healthcare professional supports deliberation throughout the process.

Six dialysis coordinators were trained in SDM, decision coaching, tailoring delivery of the intervention to patients’ needs [18] and using three different communication skills: mirroring, active listening and values clarification [19–21]. The initial training lasted two working days with a follow-up session every 6 months of one to 2 days. The dialysis coordinators were all offered supervision throughout the intervention period. Given that they were instructed to tailor the meetings to the patients’ needs, patients had from one to four meetings.

The PDA was designed to be used during and between the SDM-DC meetings. The PDA is in paper format and consists of a set of tools: a decision map, an overview of uremic symptoms, an overview of options [22–24], and the Ottawa Personal Decision Guide (OPDG) [25, 26]. More specifically, the PDA makes explicit the dialysis decision, and describes options, benefits and harms using the best available evidence. The intention is to help patients clarify their values by indicating the importance of the benefits and harms on a scale from 0 to 5. It is included in the international inventory of PDAs at https://decisionaid.ohri.ca/ where it scored 30 out of 33 on the IPDAS items. It failed to meet the criteria for: a) reporting evidence sources in the PDA; and b) two evaluation criteria which will be achieved in this study. Four videos with personal stories were available to be shown and discussed at the meetings if the patient preferred to see the reason why another patient had chosen a specific option. Each video covered one option, with a patient explaining why he or she chose that option, and how he or she weighed up the advantages and disadvantages.

### Data collection procedures

At the last SDM-DC meeting, the dialysis coordinator gave the patient the study questionnaires. Patients could: a) answer the questionnaire immediately; b) take it with them and answer it at home, then send it to the hospital by mail or bring it to their next consultation at the hospital; or c) arrange for the dialysis coordinator to call them 14 days after the last meeting to answer the questions during the call. Data from the questionnaires was entered into SurveyXact® [27]. Patient characteristics were registered in an Excel® file for all patients given the invention, whether or not they participated in the study. A consecutive sample of patients who had answered the questionnaires were asked to participate in a semi-structured interview [28]. An interview guide was developed based on the Three-Talk model [6] and adapted for each participant based on the individual’s responses to the questionnaires. The interviews were conducted in direct extension of interviews as previously described [10]. Thus, it was the same participants who contributed in both studies. The analysis for the current study is completely new, with its own data set and its own aim. The interview guide covered why the participants had answered the questionnaires as they did and participants’ reasons for their preferences for and against each dialysis modality.

### Outcome measures

Evaluation was conducted to verify that this intervention met the IPDAS evaluation criteria focused on ensuring the intervention supported the process of decision-making and achieved an outcome of a high-quality decision.

An SDM questionnaire (SDM-Q9) was used to measure patients’ perception of SDM in the clinical encounters [29]. It consists of nine statements that the participant rated on a six-point scale from ‘completely disagree’ (0) to ‘completely agree’ (5) [29]. The questionnaire has previously been used to measure SDM in dialysis choice [30] and the translated Danish version was previously validated [31]. The Danish SDM-Q9 has an internal consistency of 0.94 and the explanatory factor analysis showed a unidimensional factor [31].

The Decision quality measurement (DQM) questionnaire consists of six knowledge statements and six readiness statements. Participants answer items on the questionnaire as ‘yes’, ‘no’ or ‘unsure’. It also includes two open questions: ‘What influenced you most in making your decision?’ and ‘What was the most important consideration in making your decision?’. The second open question measured the patients’ preference. DQM is based on the Decision Quality Instrument [32] and has been used to measure SDM in dialysis choice [22]. The instrument was designed for use in clinical encounters rather than as a research instrument [33]. For this study the DQM instrument was translated into Danish through a standardized forward-backward translation [34] and face validity evaluated using cognitive interviews with patients prior to this study.

### Data analysis

All quantitative data analysis was performed using STATA® version 15 [35]. A comparison of the characteristics of the patients in the study sample and the non-study sample was conducted to determine the representativeness of the study sample. Statistical comparisons with categorical data were done by use of a Chi test or Fisher’s exact test (expected values below 5) and *p* < 0.007 was considered to be the limit of significance in accordance with the Bonferroni rule. Then for the study sample, we evaluated both the SDM-Q9 and the DQM. We compared outcomes for those who chose home-based versus those who chose hospital-based treatment. Statistical differences between the sample choosing home- and hospital-based treatment were identified using a Chi test and *p* < 0.05 was considered to be the limit of significance. The quality of the decision-making process was calculated as the mean score for each item in the SDM-Q9. To provide a total score for the SDM-Q9, a sum of all items was calculated and standardized on a scale of 0–100. Cronbach’s alpha was 0.87 and showed high internal consistency. Decision outcomes were calculated for knowledge and readiness for decision-making. The percentage of patients choosing the right answer for each knowledge statement was calculated. A total knowledge score was calculated and standardized out of 100. The readiness score was calculated by summing up all positive responses to the six readiness statements and standardizing the score out of 100. The open questions were analysed using descriptive qualitative analysis and ranked based on most common to least common comments. A concordance score [36] had not previously been calculated for dialysis choice. We concluded that deciding on a home-based treatment and choosing ‘Treatment at home’ for the preference question or deciding on a hospital-based treatment and not choosing ‘Treatment at home’ for the preference question should be considered concordant choices. A decision quality score is an association between a high knowledge score and a concordance score [36]. A decision quality score had not previously been calculated for dialysis choice, but consistent with decision quality in the ‘Hip-knee osteoarthritis decision quality instrument’ [37], it was defined as a knowledge score of > 66% combined with the concordance score.

The semi-structured interviews were transcribed and analysed using systematic text condensation [38]. Quotations from the interviews have been translated into English as accurately as possible.

### Ethical considerations

Participation in the intervention was based on consent for care and treatment. According to Danish legislation, questionnaire and interview studies are exempted from ethical approval. The Danish Data Protection Agency (jr. 1–16–02-456-16) approved data management. We obtained written consent from patients before they completed the questionnaires and qualitative interviews.

## Results

### Participant flow and characteristics

**Fig. 1:**
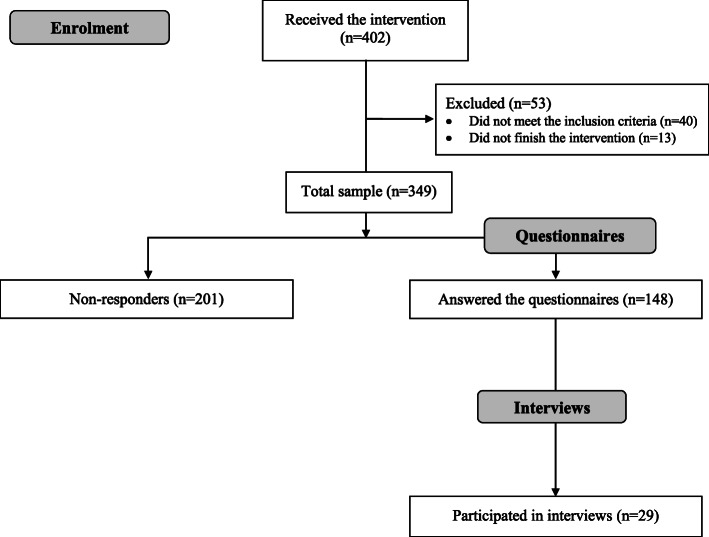
Consort flow diagram of the participants

Between October 2016 and May 2018, a total of 402 patients received the intervention (see Fig. 1). Some who were exposed to the intervention (*n* = 53; 13%) were excluded from the study because they were ineligible: 40 (10%) had chosen conservative management, and 13 (3%) did not complete the intervention because their condition improved or they had a transplant. Of the 349 eligible participants, 148 (42%) consented to participate and provided a response to the questionnaires (study sample), and 29 (8%) were invited and consented to participate in an interview. The response rate for the questionnaires varied across the four hospitals from 34 to 82%.

Among the 349 eligible participants who were exposed to the intervention (total sample), the patients’ mean age was 67 ± 14 years (mean ± sd), mean eGFR was 13 ± 4 ml/min, and 65% were male (see Table 1). One-third (30%) had participated in a kidney school. Most patients had two meetings (63%) with the dialysis coordinator (range 1 to 4).

**Table 1:** Participant characteristics^a^

Patient characteristics		The intervention sample (***n*** = 349) n (%)	The study sample (n = 148) n (%)	The non-study sample (***n*** = 201) n (%)	Chi test or Fisher’s exact test
**Hospitals**^**b**^	I	180 (52)	61 (41)	119 (59)	< 0.01
	II	53 (15)	18 (12)	35 (17)	
	III	60 (17)	49 (33)	11 (6)	
	IV	56 (16)	20 (14)	36 (18)	
**Sex**	Female	123 (35)	53 (36)	70 (35)	0.849
	Male	226 (65)	95 (64)	131 (65)	
**Age (years)**	age < 40	17 (5)	4 (3)	13 (7)	0.153^c^
	40 ≤ age < 50	21 (6)	10 (7)	11 (6)	
	50 ≤ age < 60	45 (13)	18 (12)	27 (13)	
	60 ≤ age < 70	94 (27)	36 (24)	46 (23)	
	70 ≤ age < 80	112 (32)	61 (41)	63 (31)	
	age ≥ 80	59 (17)	19 (13)	41 (20)	
**eGFR (ml/min)**	eGFR < 10	82 (23)	30 (20)	52 (26)	0.301^c^
	10 < eGFR < 20	244 (70)	110 (74)	134 (67)	
	eGFR > 20	23 (7)	8 (6)	15 (7)	
**Attended kidney school**	Yes	103 (30)	62 (42)	41 (20)	< 0.01
	No	246 (70)	86 (58)	160 (80)	
**Number of meetings with dialysis coordinator**	1	90 (26)	17 (12)	73 (36)	< 0.01
	2	215 (62)	102 (69)	113 (56)	
	3	40 (11)	27 (18)	13 (7)	
	4	4 (1)	2 (1)	2 (1)	
**Chosen option**	Home peritoneal dialysis	228 (65)	105 (71)	123 (61)	0.039
	Home haemodialysis	26 (8)	14 (10)	12 (6)	
	Dialysis at hospital	87 (25)	27 (18)	60 (30)	
	No decision	8 (2)	2 (1)	6 (3)	

^a^Data used in this table has been registered by the dialysis coordinators and is consistent with the documentation in the patients’ electronic health records

^b^The roman figures indicate each of the participating hospitals

^c^Exact age and eGFR were used to calculate the *p*-value and not the age group and eGFR group

In the study sample (*n* = 148), participants’ mean age was 68 ± 12 years, mean eGFR was 13 ± 4 ml/min, and 64% were male (see Table 1). Forty-two percent had participated in a kidney school and 69% had two meetings with the dialysis coordinator (range 1 to 3).

Those in the study sample had similar characteristics to the non-study sample in terms of sex, age and eGFR. There were more study participants who: a) had participated in kidney school before the intervention; b) chose a home-based treatment; and c) participated in at least two meetings with the dialysis coordinator.

### Decision-making process outcomes and experiences

A mean score of 86 ± 12 out of 100 on the SDM-Q9 instrument was obtained for the whole study sample (Table 2). There was no statistically significant difference between patients choosing a home- or hospital-based treatment (87 versus 83; *p* = 0.627). All items except one obtained a score above four [4.03 ± 1.09 to 4.63 ± 0.66] out of five reflecting that the participants strongly agreed with the SDM statements. The one item with a score below four [3.64 ± 1.48] was ‘The dialysis coordinator and I selected a dialysis treatment option together’. In the qualitative interviews, patients with both high and low scores on this item described the decision as their own.

**Table 2:** Quality of the decision-making process

Statements	Total (n = 148) Mean (sd)	Home-based treatment (***n*** = 121) Mean (sd)	Hospital-based treatment (***n*** = 24) Mean (sd)
1. The dialysis coordinator made it clear that a decision needs to be made	4.16 (1.10)	4.13 (1.08)	4.21(1.22)
2. The dialysis coordinator wanted to know exactly how I want to be involved in making the decision	4.03 (1.09)	4.05 (1.06)	3.83 (1.24)
3. The dialysis coordinator told me that there are different dialysis modalities for treating my kidney failure	4.63 (0.66)	4.65 (0.67)	4.46 (0.66)
4. The dialysis coordinator precisely explained the advantages and disadvantages of the treatment	4.50 (0.72)	4.52 (0.71)	4.42 (0.83)
5. The dialysis coordinator helped me understand all the information	4.47 (0.70)	4.47 (0.71)	4.50 (0.66)
6. The dialysis coordinator asked me which dialysis treatment option I prefer	4.60 (0.70)	4.63 (0.68)	4.42 (0.78)
7. The dialysis coordinator and I thoroughly weighed the different dialysis treatment options	4.44 (0.76)	4.44 (0.76)	4.42 (0.78)
8. The dialysis coordinator and I selected a dialysis treatment option together	3.64 (1.48)	3.74 (1.36)	3.17 (1.86)
9. The dialysis coordinator and I reached an agreement on how to proceed	4.28 (1.02)	4.32 (0.91)	4.13 (1.45)
**Total (standardized out of 100)**	**86.10 (12.19)**	**86.57 (11.71)**	**83.43 (14.59)**

SDM-Q9 items; scores are 0 to 5. Three patients were undecided and are included in the total sample but not in the home-based sample or the hospital-based sample

*I: You disagreed [0 out of 5] that the dialysis coordinator and you selected a dialysis treatment option together.*

*P16: No, that’s my own decision.*

*I: You agreed [5 out of 5] that the dialysis coordinator and you selected a dialysis treatment option together.*

*P7: Yes and no, I both agree and disagree. It was my own choice, but she [the dialysis coordinator] also confirmed my choice and contributed to the decision.*

### Decision quality

For DQM, the total knowledge score was 82% and the readiness score was 86% (Table 3). Those choosing home-based treatment had a higher knowledge score compared to those choosing a hospital-based treatment (84% versus 75%; *p* = 0.006). There was no significant difference between groups on readiness (87% versus 84%; *p* = 0.908). The qualitative interviews showed that some of the patients had difficulty remembering the information evaluated in the knowledge test questions.

**Table 3:** Quality of the decision

	Statements	Total (***n*** = 148)	Home-based treatment (***n*** = 121)	Hospital-based treatment (***n*** = 24)
**Knowledge items**	1. Peritoneal dialysis is a treatment that takes 30 min once a day	82%	86%	75%
	2. You need a specific room for dialysis	78%	81%	63%
	3. I can eat and drink whatever I like when I am on any type of dialysis	68%	69%	58%
	4. I can go on holiday if I am on dialysis	96%	97%	96%
	5. Dialysis is usually only needed for a few months	95%	96%	92%
	6. Home haemodialysis is suitable for people who want to take responsibility for their own treatment	74%	75%	67%
	**Total knowledge score**	**82%**	**84%**	**75%**
**Readiness items**	7. I know the options available to me	97%	98%	96%
	8. I understand the options available to me	99%	99%	100%
	9. I am aware of the advantages of each option	93%	95%	88%
	10. I am aware of the disadvantages of each option	88%	88%	83%
	11. I know how I feel about each option	73%	75%	67%
	12. I can imagine what it would be like to live with each option	68%	69%	71%
	**Total readiness score**	**86%**	**87%**	**84%**

Three patients were undecided, and they are included in the total sample but not in the home-based sample or the hospital-based sample

*P3: I am not able to remember that. I have for sure heard that it [peritoneal dialysis] is at night. But I have forgotten all about it. I have totally forgotten it.*

*I: You have trouble remembering and you bring your husband to help you to remember?*

*P3: Yes, I have trouble remembering.*

The qualitative interviews showed that the right answer for a specific patient depended on the context.

*I: In the questionnaire, you have answered that you need a specific room for dialysis.*

*P13: I know you could have dialysis in your living room, being able to sit and watch TV at the same time, and you could have dialysis in your bedroom. But the living room, no. The living room should not be a hospital. Luckily, we have more than 150 m*^*2*^*. We have three rooms ready to be used.*

The most common factors participants described as influencing the decision-making process were ‘Talking to the dialysis coordinator’ (94%), ‘Talking to your doctor’ (43%) and ‘Patient decision aid’ (39%) (Table 4). The patients who chose ‘Something else’ (9%) most often specified their relatives (e.g., their wife, husband or children). Those choosing home-based treatment were more likely to pick ‘Talking to your doctor’ (47%) compared to those choosing hospital-based treatment, who were more likely to select ‘Patient decision aid’ (38%).

**Table 4:** What influenced you most in making your decision? Quantitative responses

Total sample (n = 148)	Home-based treatment (n = 121)	Hospital-based treatment (n = 24)
Talking to the dialysis coordinator 94%	Talking to the dialysis coordinator 96%	Talking to the dialysis coordinator 96%
Talking to your doctor 43%	Talking to your doctor 47%	Patient decision aid 38%
Patient decision aid 39%	Patient decision aid 40%	Speaking to other patients/ Talking to your doctor 25%
Kidney school 25%	Something else^a^/Looking on the internet 9%	Something else^a^ 17%
Speaking to other patients 11%	Speaking to other patients 8%	Kidney school 8%
Something else^a^ 9%		

^a^‘Something else’ was most commonly relatives, either the patient’s spouse or children

The most important considerations for patients making the decision were ‘Impact on your lifestyle’, ‘Having your treatment at home’, ‘Length of time taken for each treatment’ and ‘Frequency of each treatment’ (Table 5). Those choosing home-based treatment were more likely to select ‘Having your treatment at home’ compared to those choosing hospital-based treatment, who were more likely to pick ‘Something else’. The patients who chose ‘Something else’ specified safety or security, space at home, and one stated ‘needles’. Choice of the first two statements – ‘Impact on your lifestyle’ and ‘Having your treatment at home’ – is consistent with preferences mentioned in the qualitative interviews. In the qualitative interviews, patients nearly always stated the same preferences (Table 6) as those they gave in the questionnaires. Patients who chose a home-based treatment also stated ‘Freedom’ and ‘To travel’ as preferences. Patients choosing a hospital-based treatment had different specific preferences: ‘Do not want changes at home’, ‘To be dependent upon home care’ and ‘Less work’.

**Table 5:** Most important consideration when making the decision – quantitative responses

Total sample (n = 148)	Home-based treatment (n = 121)	Hospital-based treatment (n = 24)
Impact on your lifestyle 72%	Having your treatment at home 88%	Something else 38%
Having your treatment at home 72%	Impact on your lifestyle 81%	Impact on your lifestyle 29%
Length of time taken for each treatment 20%	Length of time taken for each treatment 20%	Frequency of each treatment 25%
Frequency of each treatment 20%	Frequency of each treatment 19%	Distance to nearest dialysis unit 21%
Distance to nearest dialysis unit 11%	Distance to nearest dialysis unit 11%	Medical factors 17%
Something else^a^ 11%	Something else^a^ 6%	Unsure^b^ 13%
Medical factors 5%		Having your treatment at home 4%
Unsure^b^ 3%		

^a^‘Something else’ is chosen when the patient wishes to specify a factor that is not included in the considerations given in the questionnaire

^b^‘Unsure’ is when the patient is not sure what has been the most important consideration

**Table 6:** Most important consideration when making the decision – qualitative responses

Home-based dialysis	Hospital-based dialysis
**To be at home or not to be at the hospital** (12 sources & 18 references) *You leave home in the morning and you come back home late, then you are nearly admitted to the hospital. And I am all right. [P21]*	**Safety** (6 sources & 16 references) *I would be safer having my treatment at the hospital. [P11]*
**Transportation** (12 sources & 22 references) *It is the transportation. We do not drive ourselves. We take two different buses and it takes nearly a full hour to get out there. No, it’s a hassle. It’s a bit of a mess. [P2]*	**Not enough space at home** (5 sources & 18 references) *I simply have a lack of space. I have bought a condominium, it is 78 m*^*2*^*, but everything is sloping, so I cannot put a single 60 cm high cabinet in. [P12]*
**Freedom** (12 sources & 24 references) *That is what suits my desire for freedom best. [P3]*	**Do not want changes at home** (5 sources & 8 references) *I am not hysterical, but I like that things are in order. And I would not be able to handle having ten boxes like this standing where they really didn’t belong at all. [P16]*
**Impact on your lifestyle** (11 sources & 13 references) *That dialysis mode will change our everyday life less or our life, you could put it that way. [P20]*	**Less work** (3 sources & 10 references) *I just think that when I go to bed in the evening, I have to put that night bag on, and just need to do that and that. I do not want to do a whole lot of things before I can go to bed. [P16]*
**Less time-consuming** (9 sources & 16 references) *I don’t need to waste my time. I know, when you have retired, you have a lot of time, but I still don’t want to waste my time driving from home to the hospital. [P21]*	**To be dependent upon home care** (3 sources & 6 references) *I do not want home care nurses to come to my home and help me who just stand and do nothing. [P9]*
**Better for the body** (9 sources & 11 references) *The machine has been designed to press through him in three or four hours. That is much harder on the body than the other machine. [P22]*	
**To travel** (5 sources & 7 references) *We still want to travel as long as possible. I still drive to XX [a specific city] every time they play a home game. [P10]*	

The number of sources indicates the number of interviews in which the relevant consideration was stated, and the number of references indicates how many times the consideration was mentioned. The number in square brackets is the identification number of the patient quoted

The concordance score between chosen option and ‘Treatment at home’ showed that 89% of the patients had made a concordant decision. There was no statistically significant difference in concordance scores for patients choosing home- versus hospital-based treatment (82% versus 94%; *p* = 0.24). In terms of decision quality, 83% of patients made a high-quality decision. There were no differences between patients choosing home- versus hospital-based treatment (83% versus 83%, *p* = 0.935).

Given the low response rate, we compared the site with 83% of participants to the results for the total sample. The patients from the hospital with the highest response rate (83%) obtained a similar SDM score (87 ± 10) compared to the total study sample (86 ± 12). The patients from the hospital with the highest response rate obtained a nearly equal knowledge score (83%) and readiness score (87%) compared to the total study sample.

Given the low response rate, we compared the results from each hospital and no statistics differences was found.

## Discussion

Our study sought to examine both the SDM process and decision quality after patients with kidney failure were exposed to the SDM-DC intervention involving a PDA and meetings for decision coaching by the dialysis coordinator. Over 80% of the patients reported a high degree of SDM, were knowledgeable about options, and felt ready to make a decision. Based on an informed decision and concordance between the factors reported as influencing their decision and their chosen option, 83% of participants made a high-quality decision. An important finding was the ability for the SDM-DC intervention to be delivered within four different hospitals by six dialysis coordinators trained in decision coaching.

### Participant flow and characteristics

A lower than expected proportion of patients participated in this study. Recruitment of patients to complete the study depended on the dialysis coordinators distributing the questionnaires. When we compared the study sample with the non-study sample, we found more patients who participated in the kidney school, chose a home-based treatment and had more meetings with the dialysis coordinator. Interestingly, the hospital with the highest response rate had a higher proportion of patients participating in the kidney school before the intervention (57%) than the other hospitals (18, 29 and 39% respectively). More patients in the study sample (18%) had three meetings than patients in the non-study sample (6%), and fewer patients in the study sample (11%) had one more meeting than the non-study sample (36%). It is possible that it was easier to deliver the questionnaires to patients who had two or three meetings than patients who only had one meeting. They may have cancelled an appointment. No statistically significant differences were found between patients’ characteristics in the study sample and non-study sample for those who had only one meeting with the dialysis coordinator.

### Decision-making process outcomes and experiences

The patients exposed to the intervention experienced SDM according to the SDM-Q9 total score but had lower scores on the one item that specifically asked if the decision was shared with the dialysis coordinator. Our SDM-Q9 score of 86 was higher than that in a study of German patients considering dialysis options, who scored 73 [30]. The German study the patients did not receive either a PDA or decision coaching. The German study found variations in the SDM-Q9 score between patients choosing a home-based treatment (who scored 83) and patients choosing a hospital-based treatment (who scored 61). When used in Danish gynaecology and sport clinics, the SDM-Q9 score (82%) was more consistent with our study’s findings [31].

The item asking if it was a shared decision (‘The dialysis coordinator and I selected a dialysis treatment option together’) had the lowest score in our study, and this was lower than in the other studies using the SDM-Q9 [29, 31]. This item measures whether patients experienced their role as active, collaborative or passive [39]. The Cochrane Review of PDAs reports that patients experienced less passive roles in the decision-making process, but the review did not differentiate between active and collaborative patient roles [5]. It seems that in this study, patients experienced their role as either collaborative or active. The main finding in the previously reported qualitative evaluation of SDM-DC was that patients experienced the decision as being their own, but they were not able to make the decision without support from the dialysis coordinator and PDA [10]. There was a statistically significant difference between patients who decided on a home- or hospital-based treatment (3.74 versus 3.17 out of 5; *p* = 0.044, indicating that patients choosing a hospital-based treatment perceived their role in decision-making to be more collaborative. A similar difference in the mean score for this item was found in the German patient population making a dialysis choice: home-based treatment had a mean score of 3.93 out of 5, and hospital-based treatment had a mean score of 2.80 out of 5 [30]. It is possible that patients choosing hospital-based treatments did not feel as confident in their ability to manage their dialysis or be involved in making the decision.

### Decision quality

As expected, patients exposed to the SDM intervention had high knowledge and readiness scores. Our findings were consistent with another study that measured decision quality for dialysis choice [22]. In the previous study, baseline knowledge and readiness improved after exposure to the intervention. In our study, the knowledge score was statistically significantly lower for the patient group who decided on a hospital-based treatment, but no differences were found in the readiness score. It seems that patients who choose hospital-based treatment had fewer resources for patient involvement and may have some unmet decisional needs. Our findings revealed that patients choosing hospital-based treatment were mostly dependent upon the dialysis coordinator (96%) and desired more support from their doctor. However, talking to other patients (25%) could be one way to meet some of the decisional needs of patients who decided on hospital-based treatment, as could engaging their relatives (17%) in the decision-making process.

Concordance score and decision quality have never previously been measured for dialysis choice. In our study, 89% achieved concordant decisions, which was the same as a breast cancer study in the US [40]. In our sample, 83% of patients achieved decision quality, which was higher than a similar composite measure indicating that 56% of patients considering treatment options for hip and knee osteoarthritis achieved decision quality [37].

In contrast to other SDM interventions for dialysis choice, the SDM-DC intervention has been implemented in clinical practice, and it has so far been evaluated in relation to the two IPDAS evaluation criteria: SDM process and the decisional outcomes. Other studies evaluating SDM interventions for dialysis choice have used other measurements, making it difficult to compare the interventions. However, the data on patient characteristics shows that, of all 349 patients who received the SDM-DC intervention, 72% decided on a home-based treatment versus 25% who decided on a hospital-based treatment. Other SDM interventions for dialysis choice have shown a lower number of patients choosing a home-based treatment. A study from Spain [41] and one from the UK [24] both showed a distribution of 50/50, while a study from Australia [23] showed a distribution of 60/40. Consequently, it seems that our SDM intervention for dialysis choice meets most of the IPDAS criteria and increases the number of patients choosing a home-based treatment. This study shows only slightly different results for the SDM process and decisional outcomes between the participants choosing a home-based treatment compared to those choosing a hospital-based treatment.

### Limitations

There are three key limitations to consider when interpreting our findings. Although low response rate is common in this patient population [24, 42], the response rate was surprisingly low. The dialysis coordinators from the three hospitals with the lowest response rates reported that they did not consistently deliver the questionnaires to all of their patients. Despite the low response rate, a comparison of characteristics of participants in the study sample and the non-study sample showed that the study sample was representative. For ethical reasons, a randomized comparative study design was not an option because the intervention had been pilot tested in one of the hospitals before this study [7] and PDAs had previously been evaluated in over 100 randomized controlled trials [5]. A pre- and post-test design was rejected to minimize the burden on the vulnerable patient group involved and to increase the number of participants exposed to the intervention. Also, using patients at other hospitals in Denmark as a control group was not possible, because the hospitals were not able to identify suitable patients and did not offer patients the opportunity to participate in the decision-making process. Not having a parallel control group made it difficult to determine the effect of the SDM-DC intervention, but over 100 randomized controlled trials evaluating SDM interventions and demonstrating their effectiveness support a potential causal interpretation of our findings [5]. As an alternative, we used mixed methods to strengthen the data. Finally, given the low response rate, there is the potential for selection bias by the dialysis coordinators. Furthermore there was a potential for response bias given that the dialysis coordinators collected the study questionnaires, and patients may have responded in favour of their role in supporting decision-making. However, the knowledge test was objective, and patients had not been exposed to the questions previously.

## Conclusions

Although this study did not include a control group, over 80% of the participants exposed to SDM-DC experienced an SDM process and reached a high-quality decision. Both participants who chose home- and hospital-based treatment experienced the intervention as SDM and made a high-quality decision. Qualitative findings supported the quantitative results. Most participants described the decision to be their own choice. The two patient groups (home- versus hospital-based treatment) may have different decisional needs and may have benefited from different elements in the SDM-DC intervention. Thus, dialysis coordinators should be trained to tailor their coaching to patients’ individual needs, and future research should determine whether more specific and focused coaching is required.

## Data Availability

No additional data is available.

## Abbreviations

DQM: Decision quality measurement
IPDAS: International Patient Decision Aids Standards
PDA: Patient decision aid
SDM: Shared decision-making
SDM-DC: Shared Decision-Making and Dialysis Choice
SDM-Q9: Shared decision-making questionnaire with nine items
